# Supplementary material for: Prostate cancer survivors with symptoms of radiation cystitis have elevated fibrotic and vascular proteins in urine
Source: PLoS One. 2020 Oct 29;15(10):e0241388. doi: 10.1371/journal.pone.0241388 (PMC7595289; doi:10.1371/journal.pone.0241388)
Supplement: S1 File — After providing written informed consent, all participants completed this survey to collect information on patient demographics, prostate cancer history, radiation therapy, and bladder health. (PDF) [file pone.0241388.s001.pdf]

For Office Use Only:
*Survey Number*____________________
*Date*________________________

TÍTULO DEL ESTUDIO

Estudio de marcadores biológicos presentes en la vejiga de pacientes tratados con radioterapia pélvica

ENCUESTA para pacientes participantes

Edad: _____________ **Talla** (metros): ________________ **Peso** (kilogramos): ____________

Género: ☐ Masculino ☐ Femenino

Estado civil:
☐ Soltero ☐ Casado ☐ Separado ☐ Divorciado ☐ Conviviente ☐ Viudo

Nivel educacional alcanzado:

☐ Enseñanza Básica ☐ Enseñanza Media ☐ Técnico – Profesional ☐Licenciado - Universitario
☐ Profesional – Universitario

Actividad Física:

☐ No hago ejercicio
☐ Hago ejercicio 1,5 horas o más a la semana
☐ Hago ejercicio 5 horas o más a la semana

Tabaquismo:

☐ Nunca he fumado ☐ Yo fumaba en el pasado ☐Actualmente fumo
☐ Yo uso tabaco de mascar o para aspirar (rapé, snuff)

Si usted ha fumado, cuántos paquetes fuma usted al mes? _____________

SERVICIO DE SALUD METROPOLITANO CENTRAL
COMITE ETICO CIENTIFICO
25 ENE 2016

Alcohol:

¿Bebe alcohol? ☐ Sí ☐ No
En caso afirmativo, ¿cuántas bebidas alcohólicas bebe usted por semana (en promedio)?
____

En caso afirmativo, ¿cuántos días durante el último año has bebido 5 o más bebidas alcohólicas en un día?
____

Título: Estudio Colaborativo Internacional para la determinación de marcadores biológicos presentes en la vejiga de pacientes tratados con radioterapia pélvica
Versión 1.0
Fecha 15-01.2016

For Office Use Only:
*Survey Number*______________________
*Date*______________________________

Antecedentes Médicos:

¿Ha recibido alguna vez radioterapia? ☐ Sí ☐ No

En caso afirmativo,

a) ¿A qué edad recibió usted radioterapia? _____________

b) ¿Para qué tipo de cáncer recibió usted radioterapia?

☐ Cáncer de próstata ☐ Cáncer de vejiga ☐ Cáncer de cuello uterino
☐ Cáncer de Colo/Rectal ☐ Cáncer de ovario cáncer ☐ Otros (señalar tipo de cáncer):

c) ¿Qué tipo de radiación recibió usted?

☐ Radiación externa
☐ Braquiterapia (interna)
☐ No lo sé

¿Cuántas sesiones recibió?

	1		11 - 15		25 - 30		41 - 45
	2 - 5		16 - 20		31 - 35		> 46
	6 - 10		21 - 25		36 - 40		Yo no recuerdo

Alguna vez un médico le ha informado que usted tiene una cistitis actínica y/o hemorrágica (daño de la vejiga por radiación)?

☐ Sí ☐ No

En caso afirmativo,

a) ¿Cuántos años después de la radiación comenzaron sus síntomas? _____________

b) ¿El diagnóstico de cistitis actínica fue confirmado con una cistoscopia (examen del interior de la vejiga con una cámara)? ☐ Sí ☐ No

c) ¿Qué tratamiento (s) ha recibido para tratar los síntomas (molestias) de la cistitis por radiación? Marque todos los que corresponda.

☐ Nitrato de Plata; ☐ Aluminio ☐ Oxígeno Hiperbárico; ☐ Formaldehído / formol; ☐ Irrigación Vesical
☐ ninguno de esos ☐ no sé / no lo recuerdo

Otros: ______________

Alguna vez un médico le hizo el diagnóstico de:

a) Infecciones Urinarias Recurrentes (más de dos en un año) ☐ Sí ☐ No
b) Litiasis o Cálculo en el riñón o en la vejiga ☐ Sí ☐No
c) Cistitis intersticial / síndrome de vejiga dolorosa (IC / BPS) ☐ Sí ☐ No

SERVICIO DE SALUD METROPOLITANO CENTRAL
COMITE ETICO CIENTIFICO
25 ENE 2016

d) Prostatitis crónica / síndrome de dolor pélvico crónico (CP / CPPS)
☐ Sí ☐ No ☐ Yo soy mujer
e) Cáncer de próstata
☐ Sí ☐ No ☐ Yo soy mujer
f) Diabetes
☐ Sí ☐ No

For Office Use Only:
Survey Number_______________
Date_______________

¿Ha recibido tratamiento con algún inmunosupresor o medicamento antiinflamatorio después de haber sido irradiado(a)? Tales como:

	Tacrolimus /Cidimus/T-INMUN		Prednisona/Prednisolona		Otro:_______________
	Ciclosporina/ Sandimmun/		Dexametasona		Ninguno
	Sirolimus/Rapamune/Sirotan		Naproxeno/Eurogesic/Naprosin		Yo no recuerdo
	Ibuprofeno / Advil / Motrin/ Actron		Celebra/Celebrex		

¿Está recibiendo o ha sido tratada(o) con quimioterapia? ☐ Sí ☐ No

En caso afirmativo,

a) ¿Qué tipo de quimioterapia recibió?_______________
b) ¿Cuándo fue su último ciclo de la quimioterapia (mes + año)? _______________

SERVICIO DE SALUD METROPOLITANO CENTRAL
COMITE ETICO CIENTIFICO
25 ENE 2016

¿Con qué frecuencia suele orinar (vaciar su vejiga) en un plazo de 24 horas?

☐ Uso un catéter vesical
☐ <3 ☐ 3-5 ☐ 6-8 ☐ 9-11 ☐ 12-14 ☐ 14-16 ☐ > 16

¿Cuánto ha sufrido en las últimas 4 semanas de los siguientes síntomas de la vejiga?
Por favor, marque con una **X** *en el cuadro que mejor describa el grado de molestia por cada síntoma señalado (1 al 6). No existen respuestas correctas o incorrectas. Por favor, asegúrese de contestar todas las preguntas.*

		Nunca	una vez a dos veces al mes	una vez por semana	muchas veces a la semana	una vez al día	muchas veces al día
**1.**	Urgencia o apremio al orinar?						
**2.**	Sangre en la orina o en su ropa interior?						
**3.**	Coágulo de sangre en su orina o en su ropa interior?						
**4.**	Despertar en la noche para orinar?						
**5.**	Pérdida involuntaria de orina? (incontinencia)						
**6.**	Espasmo, dolor o molestia en la parte baja del abdomen o en la zona genital ?						
