# Supplementary material for: Prostate cancer survivors with symptoms of radiation cystitis have elevated fibrotic and vascular proteins in urine
Source: PLoS One. 2020 Oct 29;15(10):e0241388. doi: 10.1371/journal.pone.0241388 (PMC7595289; doi:10.1371/journal.pone.0241388)
Supplement: S2 File — English translation of the patient questionnaire. (PDF) [file pone.0241388.s002.pdf]

For Office Use Only:
Survey Number___________________
Date____________________________

SURVEY

Age: ______________ **Height (meters):**_________________ **Weight (kilograms):**____________

Gender: ☐**Male** ☐**Female**

Marital Status:

	Single		Married		Separated		Divorced		Living as Married

Highest education level achieved:

	Less than high school		Some college, no degree		Bachelor's Degree
	High School		Associate's Degree		Graduate or Professional Degree

Smoking Status:

	I have never smoked		I currently smoke
	I smoked in the past		I use chewing tobacco or snuff

If you have smoked, how many packs do you smoke per year? ______________

Physical Activity Status:

	I do not exercise
	I exercise 1.5 hours or more a week
	I exercise 5 hours or more a week

Do you drink alcohol? ☐**Yes** ☐**No**

If yes, how many alcoholic drinks do you have on average per week? ___________________

If yes, how many days during the last year have you drank 5 or more drinks in a day? _____

Have you ever had radiation therapy? ☐**Yes** ☐**No**

If YES, then:

a) At what age did you receive radiation therapy? _______________

b) What cancer type did you receive radiation therapy for?

	Prostate cancer		Cervical cancer		Bladder cancer
	Ovarian cancer		Colon/Rectal cancer		Other (for what cancer type): __________________________

c) What type of radiation?

	External beam radiation
	Brachytherapy (internal)
	I do not know

How many treatments did you receive?

	1		11-15		25-30		41-45
	2-5		16-20		31-35		> 46
	6-10		21-25		36-40		I do not know

Has a doctor ever told you that you have radiation/hemorrhagic cystitis (Bladder damage from radiation)? ☐**Yes** ☐**No**

If YES , then:

a) How many years after radiation did your symptoms begin? _______________

b) Has your diagnosis been confirmed with a cystoscopy (a camera was used to look inside your bladder)? ☐**Yes** ☐**No**

c) What treatment(s) have you received to treat the symptoms of radiation cystitis? *Check all that apply.*

	Silver nitrate		Formaldehyde/formalin		I don't know
	Alum		Hyperbaric Oxygen		Other:________________
	Irrigation				

PLEASE CONTINUE TO PAGE 2 OF SURVEY

For Office Use Only:
Survey Number___________________
Date____________________________

Has a doctor ever diagnosed you with:

a) Recurrent urinary tract infections (more than two in a year) ☐**Yes** ☐**No**
b) Kidney or bladder stones ☐**Yes** ☐**Noz**
c) Interstitial cystitis/Painful bladder syndrome (IC/BPS) ☐**Yes** ☐**No**
d) Chronic prostatitis/chronic pelvic pain syndrome (CP/CPPS) ☐**Yes** ☐**No** ☐**I am a women**
e) Prostate cancer ☐**Yes** ☐**No** ☐**I am a women**
f) Diabetes ☐**Yes** ☐**No**

Have you been prescribed immunosuppressant/anti-inflammatory medication since you have had radiation therapy, such as:

	Tacrolimus/Prograf		Prednisone/Prednisolone		Other:________________________
	Ciclosporin/Sandimmune		Dexamethasone		No
	Sirolimus/Rapamune/Rapamycin		Naproxen/Aleve/Naprosyn		I do not know
	Ibuprofen/Advil/Nuprin/Motrin		Celebrex		

Are you currently receiving or have you in the past received chemotherapy? ☐**Yes** ☐**No**
If YES: **a) what type of chemotherapy did you receive?** ______________________
b) When was your last chemotherapy treatment (Month + year)? ___________________

How often do you typically void (empty bladder) in a 24 hour timeframe? ☐ **Use a catheter**

	< 3		3-5		6-8		9-11		12-14		14-16		> 16

How much have you suffered from the following bladder symptoms during the <u>past 4 weeks</u>?

Please pace an x or ✔ in the box that best describes the extent to which you were bothered by each symptom during the <u>past 4 weeks</u>. There are no right or wrong answers. Please be sure to answer every question.

	Never	Once/twice per month	Once per per week	Multiple times per week	Once per Day	Multiple times per day
1. An uncomfortable urge to urinate?	☐	☐	☐	☐	☐	☐
2. Blood in your urine or underwear?	☐	☐	☐	☐	☐	☐
3. Blood clot in your urine or underwear?	☐	☐	☐	☐	☐	☐
4. Waking at night because you had to urinate?	☐	☐	☐	☐	☐	☐
5. Accidental loss of urine? (incontinence)	☐	☐	☐	☐	☐	☐
6. Spasm or discomfort in lower abdomen or genital area?	☐	☐	☐	☐	☐	☐
